# Average paraxial power of a lens and visual acuity

**DOI:** 10.1038/s41598-023-34010-4

**Published:** 2023-05-02

**Authors:** Stephen B. Kaye, Jamila Surti, James S. Wolffsohn

**Affiliations:** 1 Department of Eye and Vision Science, Institute of Life Course and Medical Sciences, William Henry Duncan Building, University of Liverpool, 6 West Derby Street, Liverpool, L7 8TX UK; 2 School of Optometry, Health and Life Sciences, Aston University, Birmingham, UK

**Keywords:** Medical research, Translational research, Applied optics

## Abstract

To provide a solution for average paraxial lens power (A_p_P) of a lens. Orthogonal and oblique sections through a lens of power $F$ were reduced to a paraxial representation of lens power followed by integration. Visual acuity was measured using lenses of different powers (cylinders of − 1.0 and − 2.0D) and axes, mean spherical equivalent (MSE) of S + C/2, A_p_P and a toric correction, with the order of correction randomised. A digital screen at 6 m was used on which a Landolt C with crowding bars was displayed for 0.3 s before vanishing. The general equation for a symmetrical lens of refractive index (n), radius of curvature R, in medium of refractive index n1, through orthogonal ($\theta$) and oblique meridians ($\gamma$) as a function of the angle of incidence ($\alpha$) reduces for paraxial rays ($\alpha \sim 0$) to $F_{n,R} \left( {\alpha ,\theta ,\gamma } \right)\left. \right|_{\alpha \sim 0} = \frac{{n - n_{1} }}{R}\cos^{2} \theta \cos^{2} \gamma$. The average of this function is $F_{n,R} \left( {\alpha ,\theta ,\gamma } \right)\left. \right|_{\alpha \sim 0} = \frac{{n - n_{1} }}{4R}$ providing a solution of $\frac{F}{4}$ for A_p_P.For central (*p* = 0.04), but not peripheral (*p* = 0.17) viewing, correction with A_p_P was associated with better visual acuity than a MSE across all tested refractive errors (*p* = 0.04). These findings suggest that $\frac{F}{4}$ may be a more inclusive representation of the average paraxial power of a cylindrical lens than the MSE.

## Introduction

The power and focal length of a lens are a function of the angle of incidence. In clinical optics, however, treatment of power has largely been limited by paraxial approximations. The search for a suitable definition of power of a non-spherical symmetrical lens (e.g., a cylindrical lens) has a long history in medical optics^1–5^. Measurement of power away from the principal meridian (curvital and torsional power) has also depended on further approximations, such as the exclusion of the square of the sag height when calculating power of a lens^1^. Use of this non-constant approximation, however, may lead to incorrect assumptions, for example, that two equivalent orthogonal (right angle) cylinders are precisely equivalent to a spherical lens^1^. Although the spherical equivalent or more commonly the mean spherical equivalent (MSE) remains a useful scalar term for the average paraxial representation of lens power in the individual case, Harris first suggested that there may be other scalar terms for the ‘spherical equivalent’ and proposed the term nearest equivalent sphere (NES)^3^. In support of this, it has been shown that there are other representations of the average power of a lens if the square of the sag height is not excluded when calculating the radius of a lens^1^.

One of the problems with the current formula used for the MSE, that is, $S + \frac{C}{2}$, is that it does not include or represent non-orthogonal oblique rays; it is entirely based on orthogonal sections^1^. Naeser and Hjortdal referred to this in the context of ‘for any meridian of a spherocylinder, the combined spherical equivalent, net curvital and net torsional powers, direct incoming wavefronts into the two orthogonal focal lines’^5^. It is not uncommon for a lens to be tilted to the incident rays with oblique rays becoming more dominant as the lens is further tilted. The analysis of oblique or torsional rays has been discussed in detail by many authors including Pascal^4^, Naeser and Hjortdal^5^, Beldowke^6^, Keating^7,8^, Harris^9,10^, Goldstein^11^ and Bennet^12^ amongst many others. In particular, Harris, has provided solutions to the effective power of a tilted lens as power vectors and as a general tilt matrix^13,14^.

Although both orthogonal rays away from the principal meridian and oblique rays do not come into focus at the same point as meridional rays, they are still likely to have an effect on vision. Inclusion of orthogonal non-meridional rays is the basis of the MSE which does not weight meridional rays in derivation of the average. Similarly, although the contribution of oblique rays to vision is unclear, it would be incorrect to exclude them in the quantification of a thin lens system^5–8^.

In this article, solutions to the representation of power as a scalar quantity were developed and used to provide a paraxial representation of the average power of a lens. A section through a lens was first considered, followed by rotation of the section through orthogonal and non-orthogonal oblique meridians^1^. The curves resulting from orthogonal and oblique sections were considered as 2 dimensional surfaces. A function to model these sections was further developed to provide a solution for average paraxial power (A_p_P) of a lens, which included non-orthogonal oblique rays. The resultant equation for the A_p_P was then compared to the current definition for the MSE based on a clinical study.

## Methods

### Power of a lens

**Figure 1:**
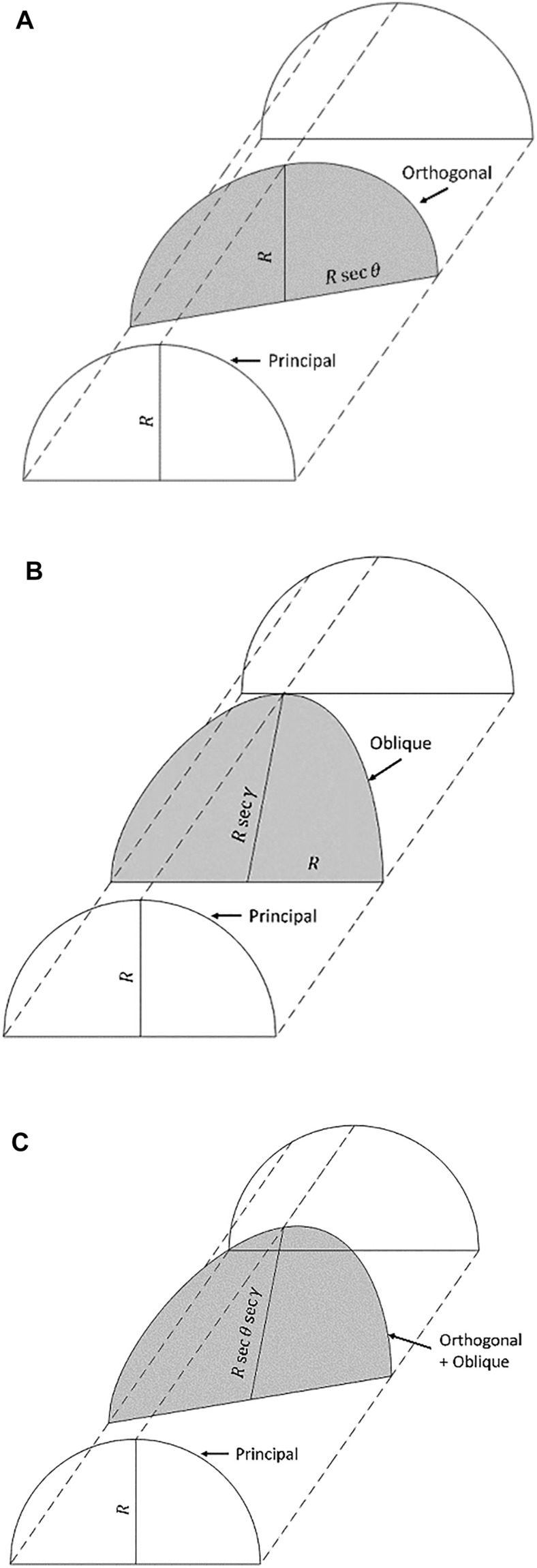
Sections through a lens cylinder. Principal meridian of radius ($R$). (**A**) Orthogonal, $R\sec \theta$, (**B**) oblique, $R\sec \gamma$ and (**C**) Orthogonal-oblique section $R\sec \theta \sec \gamma$.

A section through a lens cylinder (Fig. 1) of radius $R$ in the principal meridian, the orthogonal and non-orthogonal oblique sections at angles $\theta$ and $\gamma$ away from the principal meridian, result in elliptical sections with major radii (semi-diameters) $R\sec \theta$ and $R\sec \gamma$, respectively. The respective angles subtended are within the intersecting plane rather than the normal to the surface in 3D space. That is, it is the projection onto the 2D plane (or viewed as the ray of light travelling through the plane itself).

**Figure 2:**
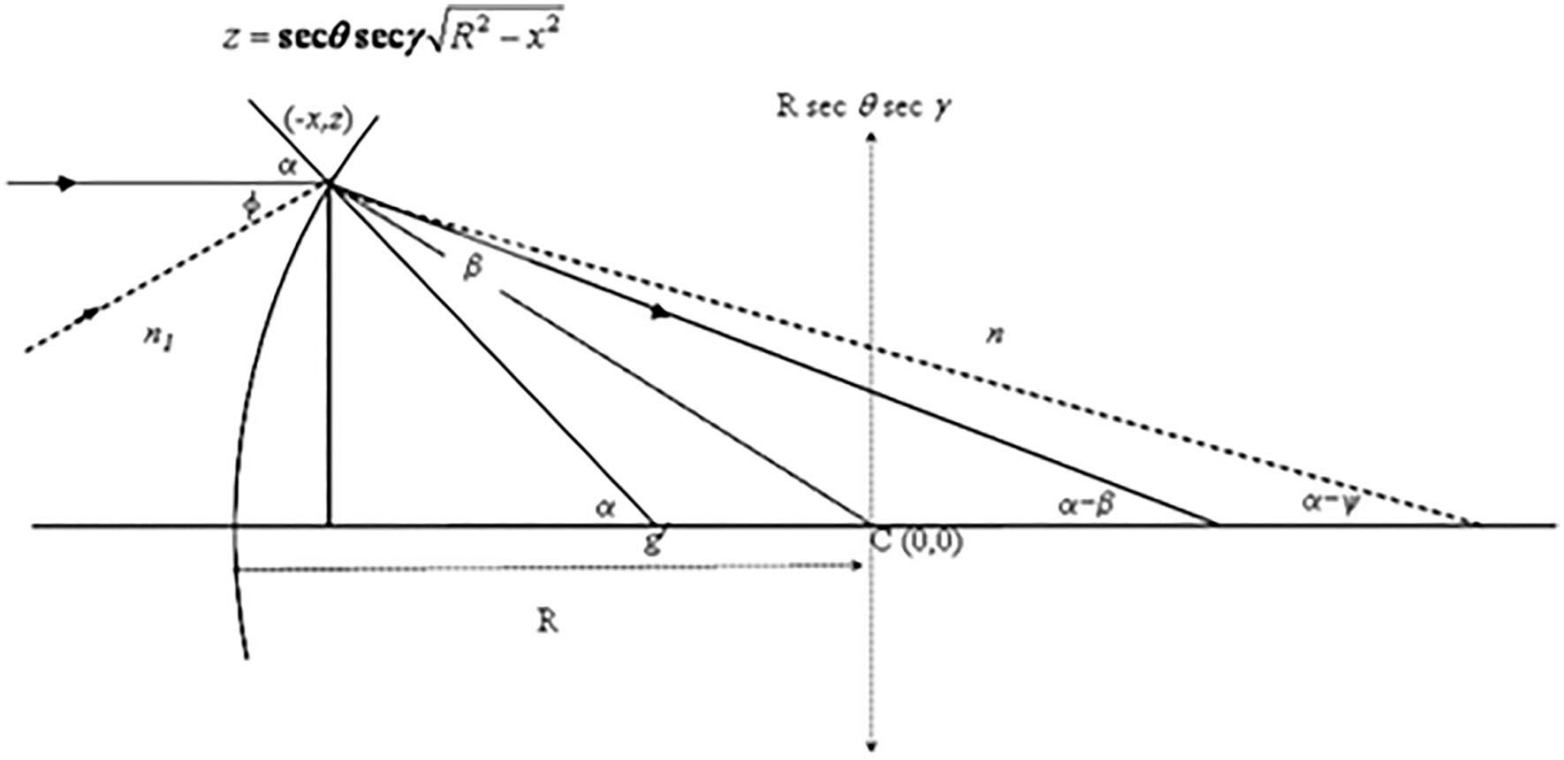
A ray travelling through a section of a lens segment of refractive index $n$, (in medium $n_{1}$) and radius $R$, with origin at $C = \left( {0,0} \right)$ of a Cartesian coordinate system with horizontal and vertical coordinates $x$ and $z$ respectively. α is the angle of incidence from a distant object ($\phi = 0$) to the normal passing through g′ and β is the angle of refraction. The angle subtend on the axis is (α–β). For light from a near object is $\phi > 0$, and the angle subtended on the axis is (α–ψ)^1^. For a symmetrical lens e.g., a cylinder, there is rotational symmetry so that both $\theta$ and $\gamma$ are periodic.

A ray travelling medium $$ n_{1}$$, into and through a section of a lens segment of refractive index $$n$$, focal length ($$f$$) and back vertex power ($$F$$), (Fig. 2). It has been shown that a section of the lens defined as an intersection of the lens with a plane (Fig. 2) can be represented by the following equation for an ellipse,

$$ \frac{{x^{2} }}{{R^{2} }} + \frac{{z^{2} }}{{R^{2} \sec^{2} \theta \sec^{2} \gamma }} = 1 $$

where $\theta$ is the angle of rotation of the intersecting plane about the $z$-axis and $\gamma$ is the angle of rotation of the intersecting plane about the $x$-axis. In the principal meridian, $\theta = \gamma = 0$ and the section is circular of radius $R$.^1^ A ray parallel to the axis of the lens ($x$-axis in Fig. 2) subtends an angle of incidence, $\alpha$ with the normal to the curve within the intersecting plane at a point $z$ where $z = \sec \theta \sec \gamma \sqrt {R^{2} - x^{2} }$,

$$ \delta ^{\prime} $$

### Reduced focal length for a distant object

The back vertex focal length in medium $n$ (reduced focal length), is $\frac{f}{n}$ with $\frac{n}{f}$ the back vertex power (*F*), a measure of the change in vergence which that surface imposes on light rays. Parallel light (distant object, $\phi = 0$) is used as the reference point given that the incident light will have zero vergence so that the vergence after passing through the surface will be equivalent to the power of that surface.

It has previously been shown^1^ that,

1 $$ F_{n,R} \left( {\alpha , \theta , \gamma } \right) = \frac{n}{R}\left\{ {\frac{{\tan \left( {\alpha - \beta } \right)\sqrt {\sec^{2} \theta \sec^{2} \gamma \tan^{2} \alpha + 1} }}{{\sec^{2} \theta \sec^{2} \gamma \tan \alpha - \tan \left( {\alpha - \beta } \right)\left( {1 - \sqrt {\sec^{2} \theta \sec^{2} \gamma \tan^{2} \alpha + 1} } \right)}}} \right\} , $$

where, the maximum angle of incidence (that is a marginal ray) is from Fig. 2,

$$ \tan \alpha_{max} = \frac{Y}{{\sqrt {R^{2} - Y^{2} } \sec \theta \sec \gamma }} . $$

It has also been shown that the same procedure can be used to determine the focal length or power for any defined surface and be used to determine the average power of any defined surface^15^.

For paraxial rays, $\beta = \frac{n1}{n} \propto$, $\sin \alpha \sim \alpha$, $\tan \alpha \sim \alpha$, $\cos \alpha \sim 1,$ $tan^{2} \alpha \to 0$ and Eq. (2) reduces to

2 $$ F_{n,R} \left( {\alpha ,\theta , \gamma } \right) = \frac{n}{R}\frac{{\alpha \left( {1 - \frac{{n_{1} }}{n}} \right)}}{{\alpha \sec^{2} \theta \sec^{2} \gamma }} = \frac{{n - n_{1} }}{R}\cos^{2} \theta \cos^{2} \gamma $$

Equation (2) can be used to determine the power of any particular section through a lens^1^.

### Average power

The current formula used to derive the average power or MSE for a lens cylinder only includes orthogonal rays or sections ($\gamma = 0$) that is, $F_{n,R} \left( {\alpha ,\theta ,0} \right) = \frac{{n - n_{1} }}{R}\cos^{2} \theta$.^1^

The MSE has been shown to be derived from the average of a function through orthogonal meridians^1^, that is, $F_{n,R} \left( {0,\theta ,0} \right)$ which from symmetry considerations is given by

3 $$ F_{n,R} \left( {\alpha ,\theta ,\gamma } \right)\left. \right|_{\alpha = \gamma = 0} = \frac{2}{\pi }\mathop \smallint \limits_{0}^{{\frac{\pi }{2}}} \frac{{n - n_{1} }}{R}\cos^{2} \theta d\theta = \frac{2}{\pi }\frac{{\pi \left( {n - n_{1} } \right)}}{4R} = \frac{{n - n_{1} }}{2R} , $$

$F$ therefore, is half the power in the principal meridian $F\left( 0 \right) = \frac{{\left( {n - n_{1} } \right)}}{R}$.

Thus, the average approximated power of a cylindrical lens through orthogonal meridians is *half* of the lens power in its principal meridian^1^. This is the justification for using the formula, $S + \frac{C}{2}$, for calculation of the MSE for a spherocylinder combination of lenses. Inclusion of non-orthogonal oblique meridians requires a further average over non-orthogonal oblique meridians for equation^5^ as follows.

For paraxial rays, the average through all orthogonal and non-orthogonal oblique meridians (rotational symmetry $0$ to $\frac{\pi }{2}$) is

4 $$ \begin{aligned} F_{p} & = \frac{4}{{\pi^{2} }}\mathop \smallint \limits_{0}^{{\frac{\pi }{2}}} \mathop \smallint \limits_{0}^{{\frac{\pi }{2}}} \frac{{n - n_{1} }}{R}\cos^{2} \theta \cos^{2} \gamma d\theta d\gamma = \frac{4}{{\pi^{2} }}\left[ {\frac{\theta }{2} + \frac{\sin 2\theta }{4}} \right]_{0}^{{\frac{\pi }{2}}} \left[ {\frac{\gamma }{2} + \frac{\sin 2\gamma }{4}} \right]_{0}^{{\frac{\pi }{2}}} , \\ & = \frac{4}{{\pi^{2} }}\frac{{n - n_{1} }}{R}\left( {\frac{\pi }{4} + \frac{\sin \pi }{4} - 0} \right)\left( {\frac{\pi }{4} + \frac{\sin \pi }{4} - 0} \right) = \frac{{n - n_{1} }}{4R} \\ \end{aligned} $$

Note from the above that $$F_{p} = \frac{F}{4}$$.herefore, the A_p_P through orthogonal and non-orthogonal oblique meridians would be $$\frac{F}{4}$$. This suggests that for a spherocylindrical lens combination (S/C$$\times a$$), after transforming into crossed-cylinder form i.e., the $$ C_{1} x a$$/$$C_{2} x a_{ \pm 90}$$, A_p_P could be represented by $$\frac{{C_{1} }}{4} + \frac{{C_{2} }}{4} $$ that is $$\frac{{C_{1} + C_{2 } }}{4}$$ rather than $$C_{1} + \frac{{(C_{2} - C_{1)} }}{2} $$. For example, $$+ 2/ + 2x90$$ or $$+ 4/ - 2x180$$ or in cross cylinder form $$+ 2x180/ + 4x90$$ has a MSE of + 3 whereas A_p_P using equation^7^ would be $$0.5 + 1 = 1.5D $$ rather than $$+ 3.00D$$.

Note, that the general geometric (non-paraxial) solution for the average power for a symmetrical lens including orthogonal and non-orthogonal oblique meridians has been shown to be approximately $\frac{1}{3}$ of the power of the lens in its principal meridian^1^. A solution to the average power of a section of any defined surface i.e., without the requirement for symmetry has been provided^15^.

### Clinical study

Fifteen young adults (average ± standard deviation 22.0 ± 6.5 years, 3 males, 12 female) with no history of ocular disease or injury, normal binocular vision (no history of strabismus or motility disorders), having less than 0.50D of astigmatism (to ensure the habitual adaption to uncorrected astigmatism is minimised^16^) and who had an acuity of 0.0 LogMAR or better in both eyes were recruited. The study was approved by the Aston University Research Ethics Committee, all subjects gave their informed consent to take part and all methods were performed in accordance with the relevant guidelines and regulations.

**Figure 3:**
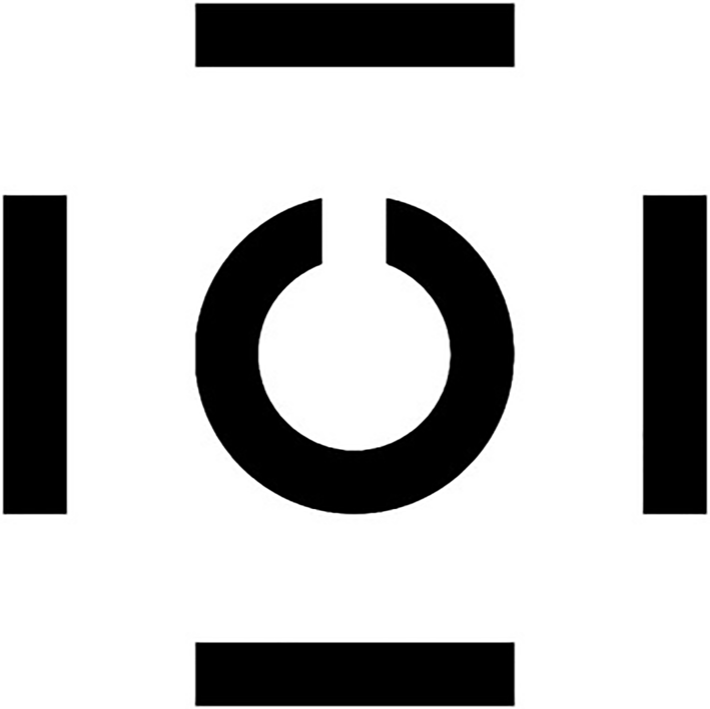
The target stimulus—Landolt C with crowding bars. This appeared at the centre of the screen in one of four orientations (right, left, up, or down), gradually changing size according to performance. Subjects were asked to report the perceived position of the gap in the C.

Subjects were simulated with different levels of a refractive errors, using wide aperture (25 mm) cylindrical lenses of different powers and axes, including − 1.00 × 90, − 1.00 × 180, − 2.00 × 90, and − 2.00 × 180 in a randomised order, in a trial frame along with lenses that corrected their refractive error, the MSE (S + C/2), the A_p_P ($\frac{{C_{1} + C_{2} }}{4}$) and full toric correction using the opposite cylinder (oriented in the same direction as the simulating lens) also in a randomised order. Visual acuity was measured monocularly, using an automated visual acuity test, developed by Wolffsohn Research (https://www.wolffsohnresearch.com/; Belfast, UK). The chart was presented on a digital screen placed 6 m away and displayed a Landolt C with crowding bars (Fig. 3), for 0.3 s before vanishing, after which subjects had unlimited time to determine the orientation of the gap in the C whether that be right, left, up or down, as part of a four-alternative forced-choice task and if uncertain, were encouraged to guess.

The target started off as a 0.3 logMAR letter size, decreasing if the response is correct and increasing if wrong, requiring 3 reversals to find the final acuity level. The final acuity was determined, based on the percentage of times the response was correct at each letter size, to the nearest 0.01logMAR.

Once subjects felt comfortable and had practiced the task, acuity was measured with central gaze first. This was repeated twice, for each level of correction, to calculate an average level of acuity. Subjects were then instructed to look at a target 30 degrees off centre horizontally to the left (nasally) and to determine the orientation of the target. Testing for peripheral vision was also repeated twice to calculate an average level of acuity. Measurements taken for the off-centre gaze (peripheral vision) were only possible with the powers − 1.00 × 90 and − 1.00 × 180 as any power higher distorted the view and the target was unable to be seen in the subject peripheral gaze.

### Statistical analysis

Parametric statistics were applied as the data were not significantly different from a normal distribution (Kolmogorov–Smirnov test *p* > 0.05). A between factors (cylinder levels: − 1.00 or − 2.00; axis 90/180; correction: traditional MSE, A_p_P, toric correction; and repeats) repeated measures analyses of variance (ANOVA) were conducted for central vision. A second repeated measures between factor ANOVA were conducted between central and peripheral vision with a cylinder level of − 1.00D with other factors (axis: 90/180; correction: traditional MSE, A_p_P, toric correction; and repeats). Student t-tests were used to determine the differences between correction approaches. A p-value of less than 0.05 was considered statistically significant and a correction made for multiple tests.

## Results

### Central vision

**Figure 4:**
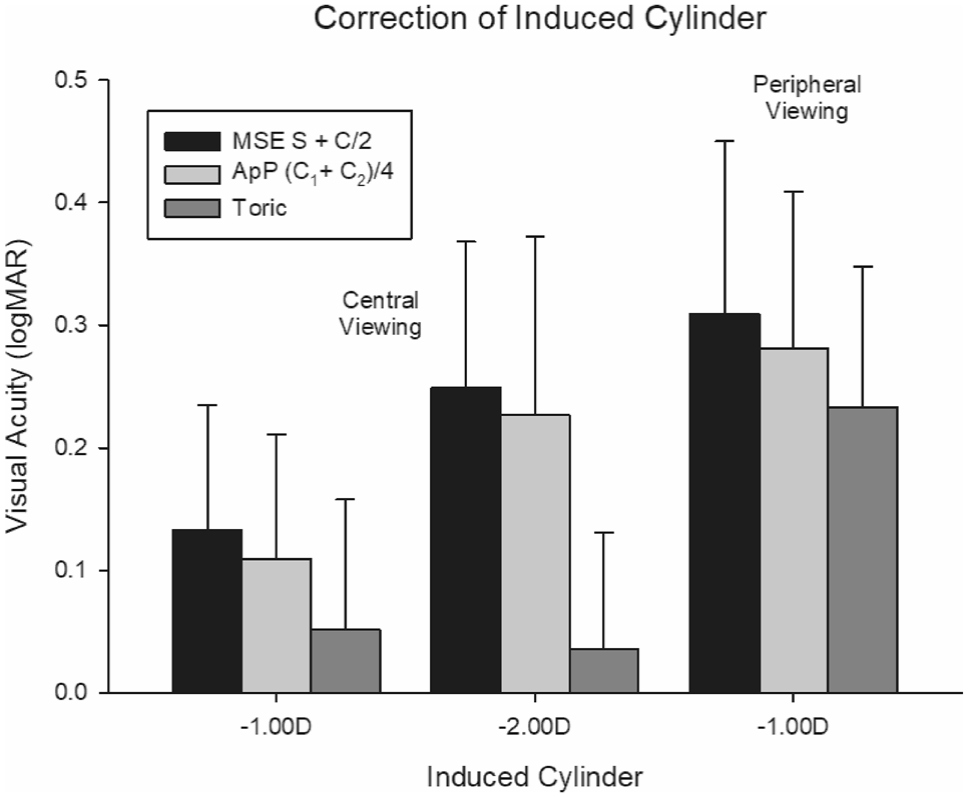
Acuity with central and peripheral target location observed with an induced cylinder and correction of MSE, ApP or toric correction of opposite power. Error bars = 1 S.D. N = 15.

For central viewing, better acuity levels were achieved with lower induced cylinders (F = 42.85, *p* < 0.001) and with the toric correction compared to either MSE and or A_p_P (F = 64.52, *p* < 0.01). There was a significant interaction between these factors (F = 13.17, *p* < 0.01) that is, the difference in the reduction in acuity increased as the refractive error increased (Fig. 4). There was no difference in visual acuity between the two orientations of the induced cylinders i.e., 90 and 180 (F = 0.07, *p* = 0.80), or with repetition (F = 3.44, *p* = 0.09), that is, there was no learning effect. For central viewing, there was a significant difference between MSE and A_p_P across all refractive errors, with the A_p_P associated with a better visual acuity (*p* = 0.04) (Fig. 4). The toric correction gave better visual acuity across all refractive errors than either the MSE or A_p_P (*p* < 0.001).

### Peripheral vision

For peripheral vision, acuity levels were lower than central vision (F = 92.77, *p* < 0.01; Fig. 4), and the differences with correction approach (F = 23.87, *p* < 0.01) were still evident, with no difference in the induced cylinder axis (F = 3.07, *p* = 0.10), or with repetition (F = 0.29, *p* = 0.60). There was a significantly worse acuity with either MSE or A_p_P corrections, compared to a toric correction (with the opposite cylinder), but this was less evident with peripheral (*p* = 0.03) than central vision (*p* < 0.01). For peripheral vision, there was no significant difference between the MSE and A_p_P across the cylinder axes (*p* = 0.17, Fig. 4).

## Discussion

Although lacking sensitivity, a scalar measure of refractive power such as the MSE is commonly used to compare or evaluate refractive powers. The average paraxial power of a lens has until now, been based only on orthogonal sections producing the familiar F/2 or for a spherocylinder combination of lenses, $S + \frac{C}{2}$, commonly referred to as the MSE. Both the MSE and A_p_P provide a scalar value i.e., not affected by direction and as such, both are representative of a spherical equivalent or more correctly a NES.

In this study a formula for the A_p_P of a lens that includes both orthogonal and oblique rays was derived. We have shown that if oblique sections or rays are included, then the A_p_P across a lens is F/4 or for spherocylinder, $\frac{{C_{1} + C_{2} }}{4}$. These findings suggest that a lens cylinder of power $C$ in the principal meridian, has a smaller average power than previously considered, that is, of $\frac{C}{4}$ rather than $\frac{C}{2}$. The average (paraxial) power of a spherical lens is the power of the lens itself and these findings apply to a symmetrical lens cylinder. The approximation of the power of a lens (geometric optics) has been addressed^1^. It is important to note, that the conversion from cross cylinder form to S/C × A, rests on the incorrect assumption that the shape formed by the intersection of two equal right-angle cylinders is a sphere, when it is in fact a bicylinder or a Steinmetz solid and not a sphere. For example, if a subject is refracted using cylinders and is found to have an error of + 2.00 × 90 and + 4.00 × 180, then the average paraxial power (A_p_P) is + 0.50 + 1.00 = 1.50D. The confusion arises if the assumption is made that + 2.00 × 90 and + 4.00 × 180 equate to a sphere of + 2.00 and a cylinder of + 2.00. It may well be that the subject does have a spherical error of + 2.00 but this cannot be assumed from two cross-cylinders, i.e., two equal cross cylinders do not necessarily equal a sphere.

Although debated, it has been reported that cylinders provide central blur that is not much different from the MSE^17,18^ but at low blur strengths relevant to refraction, astigmatism affects visual acuity more than defocus and that the degree of blur from cylinders, is dependent on the axis of the cylinder^19,20^.

It is important to note that in the derivation of the MSE and A_p_P, the average does not give different weights to meridional rays nor are limits imposed on the extent of the orthogonal or oblique rays. As information develops to quantify the relative contribution of rays away from the principal median to vision (central and peripheral) it will then be possible to weight or restrict rays in the formulation of the average power of a lens.

Which of these systems provide a better scalar measure of power for visual blur, that is, to what extent does MSE ($S + \frac{C}{2}$) and A_p_P ($\frac{{C_{1} + C_{2} }}{4})$ affect blur for a person with best corrected acuity using a spherocylinder correction of $\frac{S}{C} \times a$? To address this, two solutions for the mean power of a lens, MSE and A_p_P, were compared in a clinical study, measuring both central and peripheral (30 degrees) visual acuity. Both central and peripheral vision reduced as the refractive error increased but that central vision was significantly less affected using the A_p_P as a correction rather than the MSE; hence A_p_P led to a significantly less reduction in central acuity than the MSE. Central visual acuity is not limited to only orthogonal rays as even with an acuity of 0.00 logMAR, the visual angle subtended is 60 s of arc. In addition, a difference was demonstrated between the reversal of the induced toric and MSE and F/4, indicating that the methodology was sensitive to detect change due to refractive correction. Reversing the toric didn’t fully restore distance acuity, presumably due to the aberrations and reflections from the additional two trial lenses.

There are limitations to the study. The derivation of the average power of a lens A_p_P does not differentially weight meridional rays or rays close to the principal meridian. The same however, applies to the MSE. Further work may seek to modify or weight such averages particularly if the effect of oblique and non-meridional orthogonal rays on vision is better elucidated. It has been suggested that low frequency contrast detection may determine the position of the gap in a Landolt C^21^, but this is still more consistent than traditional capital letter charts and the participants acted as their own control. Further work is needed to explore additional refractive errors, including cylinders of higher powers and at multiple axes away from 90 and 180 degrees and using an eye tracker to help monitor gaze. For peripheral acuity 30 degrees eccentricity was explorated. This may account for the absence of a difference between MSE and A_p_P for peripheral acuity, as measurements taken for off-centre gaze (peripheral vision) at 30 degrees were only possible with the powers -1.00 × 90 and -1.00 × 180, as any power higher distorted the view and the target was unable to be seen in the subject peripheral gaze. Smaller angles, therefore, would be preferable, that is, 2.5, 5 and 10 degrees as this will enable higher powers with less distortion than occurred at 30 degrees. In addition, it would be of interest to tilt the lens systems (toric, MSE and A_p_P) from zero through to 30 degrees for central vision as this would increase the contribution of oblique rays.

In this study a solution for the A_p_P of a symmetrical lens that includes oblique rays was provided. MSE and A_p_P was then compared in a clinical study, measuring both central and peripheral visual acuity. The method for A_p_P led to lesser reduction in central visual acuity compared to using MSE. Subject to further studies, this would suggest that a A_p_P of $\frac{{C_{1} + C_{2} }}{4}$ may provide a better scalar measure than the traditionally clinically use MSE of $S + \frac{C}{2}$ and the A_p_P, therefore, may be a better approximation of the NES than the MSE. Although further investigation is needed, these findings would suggest as an example, that if a person is unable to tolerate a full toric spectacle correction, then using the A_p_P i.e., half the MSE, may provide a better level of visual acuity than the MSE.

## Data Availability

The datasets used and/or analysed during the current study available from the corresponding author on reasonable request.
