# Publisher Correction: Average paraxial power of a lens and visual acuity

**DOI:** 10.1038/s41598-023-36338-3

**Published:** 2023-06-07

**Authors:** Stephen B. Kaye, Jamila Surti, James S. Wolffsohn

**Affiliations:** 1 Department of Eye and Vision Science, Institute of Life Course and Medical Sciences, William Henry Duncan Building, University of Liverpool, 6 West Derby Street, Liverpool, L7 8TX UK; 2 School of Optometry, Health and Life Sciences, Aston University, Birmingham, UK

Correction to: *Scientific Reports* https://doi.org/10.1038/s41598-023-34010-4, published online 02 May 2023

The original version of this Article contained an error in Figure 1, where panel (A) was a duplication of panel (B). The original Figure 1 and accompanying legend appear below.

**Figure 1:**
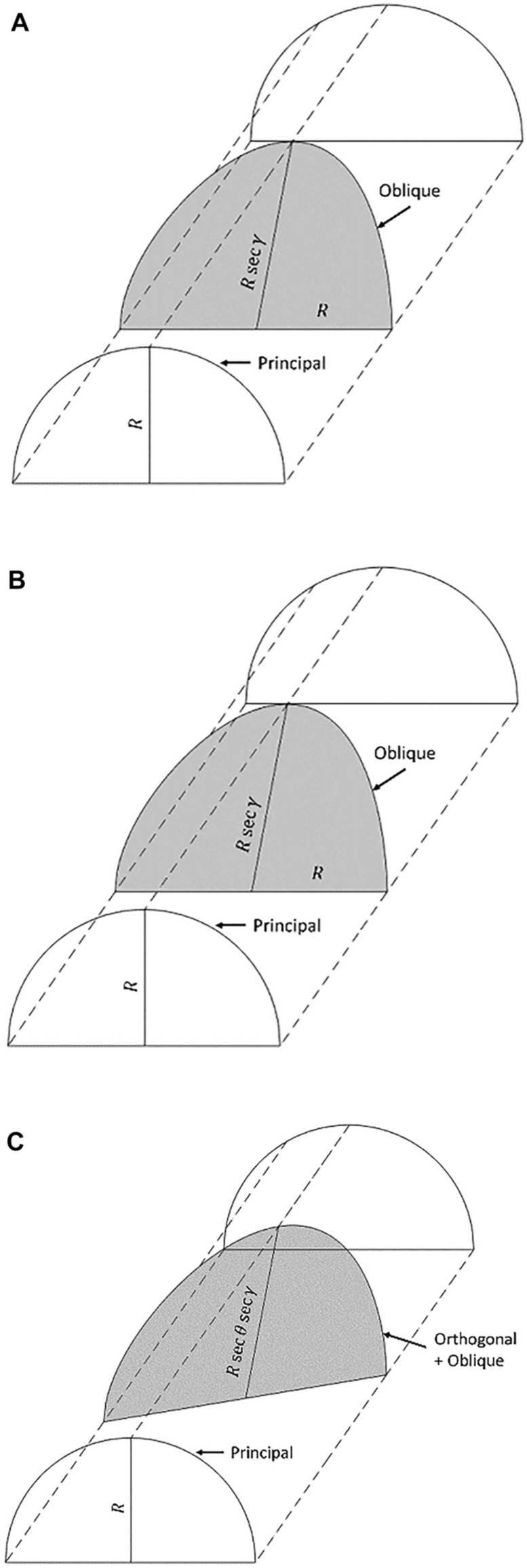
Sections through a lens cylinder. Principal meridian of radius (*R*). (**A**) Orthogonal, *R* sec θ, (**B**) oblique, *R* sec *γ* and (**C**) Orthogonal-oblique section *R* sec *θ* sec *γ.*

The original Article has been corrected.

